# Power of Nature in the Cure of Disease

**Published:** 1871-05

**Authors:** 


Power of Nature in the Cure of Disease.—There seems to be a large class of practitioners who entirely ignore the power of nature in the cure of disease, and of its ability to relieve the system from the effects of poisons. We often receive, and also find recorded in the journals, vaunted cures of tetanus by some article of the materia medica which happened to be administered at the time, the reporter overlooking the fact that the same article has over and over again failed in other cases; and even that some more potent measure, such as amputation of the injured limb, may have been employed at the same time. This last, if it fails to cure in many instances, still may exert too important an influence to be entirely ignored. It should further be remembered that more than fifty medicinal agents of the most varied and even contrary action have been claimed to have effected cures of this affection, and yet the mortality from it continues undiminished.

We likewise have received accounts of numerous cases of opium poisoning supposed to be cured by belladonna, and of belladonna poisoning believed to be cured by opium, and of strychnia poisoning cured by various articles. Now, without denying the possible antagonism of opium and belladonna, the fact must not be overlooked that both of these poisons, as well as strychnia, snake-poison, &c., may be eliminated from the system by the natural emunctories, especially by the kidneys, and that if the patient can be kept alive long enough to enable the emunctories to eliminate those poisons, recovery from their effects will take place. Without wishing to discourage the use of antidotes, we desire to impress upon our readers that the great object should be to maintain life as long as possible, so as to afford time for the eliminative process to be accomplished, and that in order to duly determine the effects of supposed antidotes the powers of nature should not be ignored, but that an effort be made to determine the precise agency of these two powers in every case of recovery, so that the real value of the so-called antidotes may be the more certainly determined.

